# Identifying Modulated Functional Connectivity in Corresponding Cerebral Networks in Facial Nerve Lesions Patients With Facial Asymmetry

**DOI:** 10.3389/fnins.2022.943919

**Published:** 2022-06-27

**Authors:** Hao Ma, Yu-lu Zhou, Wen-jin Wang, Gang Chen, Qing Li, Ye-chen Lu, Wei Wang

**Affiliations:** ^1^Department of Plastic and Reconstructive Surgery, Shanghai Ninth People’s Hospital, Shanghai Jiao Tong University School of Medicine, Shanghai, China; ^2^MR Collaborations, Siemens Healthineers Ltd., Shanghai, China; ^3^Wound Healing Center, Ruijin Hospital, Shanghai Jiao Tong University School of Medicine, Shanghai, China

**Keywords:** facial nerve, cerebral plasticity, facial asymmetry, functional connectivity, interhemispheric inhibition, fMRI

## Abstract

Facial asymmetry is the major complaint of patients with unilateral facial nerve lesions. Frustratingly, although patients experience the same etiology, the extent of oral commissure asymmetry is highly heterogeneous. Emerging evidence indicates that cerebral plasticity has a large impact on clinical severity by promoting or impeding the progressive adaption of brain function. However, the precise link between cerebral plasticity and oral asymmetry has not yet been identified. In the present study, we performed functional magnetic resonance imaging on patients with unilateral facial nerve transections to acquire *in vivo* neural activity. We then identified the regions of interest corresponding to oral movement control using a smiling motor paradigm. Next, we established three local networks: the ipsilesional (left) intrahemispheric, contralesional (right) intrahemispheric, and interhemispheric networks. The functional connectivity of each pair of nodes within each network was then calculated. After thresholding for sparsity, we analyzed the mean intensity of each network connection between patients and controls by averaging the functional connectivity. For the objective assessment of facial deflection, oral asymmetry was calculated using FACEgram software. There was decreased connectivity in the contralesional network but increased connectivity in the ipsilesional and interhemispheric networks in patients with facial nerve lesions. In addition, connectivity in the ipsilesional network was significantly correlated with the extent of oral asymmetry. Our results suggest that motor deafferentation of unilateral facial nerve leads to the upregulated ipsilesional hemispheric connections, and results in positive interhemispheric inhibition effects to the contralesional hemisphere. Our findings provide preliminary information about the possible cortical etiology of facial asymmetry, and deliver valuable clues regarding spatial information, which will likely be useful for the development of therapeutic interventions.

## Introduction

Facial nerve lesions can be caused by various forms of trauma and iatrogenic treatments, and are the second commonest cause of facial paralysis after Bell’s palsy (Baugh et al., 2013). After a facial nerve lesion, patients usually have facial asymmetry, of which oral commissure asymmetry is one of the main manifestations. In clinical practice, patients have different levels of oral commissure asymmetry. The bilateral facial muscles generate an optimal balance of tension to maintain facial symmetry in healthy individuals (Linstrom, 2002; Trotman et al., 2020). In our previous study, we reported that patients with unilateral facial nerve lesions often have hypertonic facial muscles on the unaffected side (Ma et al., 2022). The dysfunction of unaffected facial muscles plays an important role in facial asymmetry at rest as well as exaggerated, excessive facial motion when smiling. However, the underlying mechanism and precise etiology about the dysfunction have not been well described. We believe that neuroimaging is a vital first step to begin to understand this field.

In our clinical observations, patients with homogeneous etiologies show high heterogeneity in asymmetry outcomes (Chen et al., 2017; Ma et al., 2022). A recent study has demonstrated that facial nerve lesions induce widespread cerebral plasticity, including changes in cortical reorganization and functional connectivity, which correlate with clinical severity (Klingner et al., 2012; Song et al., 2017; Ma et al., 2021a,b). There is also evidence to suggest that cerebral plasticity contributes to facial muscle dysfunction (He et al., 2014; Xiao et al., 2015; Ma et al., 2021b). Deefferentation from the cerebral to the peripheral facial nerve leads to excessive activation of primary motor areas, resulting in enhanced facial activity (Xiao et al., 2015; Ling et al., 2021). Furthermore, it appears that cortical reorganization after motor deefferentation contributes to facial asymmetry (Klingner et al., 2021). As peripheral neuropathy occurs within the top-down pathway, sensorimotor adaptation is triggered by a mismatch between the desired symmetry and excessive activation (Klingner et al., 2014). Many studies have demonstrated the crucial role of sensorimotor adaptation on mismatch during facial motor tasks (Hu et al., 2015; Klingner et al., 2019; Ma et al., 2021b). In contrast, static facial asymmetry is the result of an imbalance of bilateral facial muscle tension. However, the precise link between cerebral plasticity and oral asymmetry in the resting state has not yet been systematically explored. The main goal of the present study was to investigate whether resting-state cerebral function is associated with resting-state oral asymmetry.

In our study, 22 patients with unilateral facial nerve lesions underwent functional magnetic resonance imaging (fMRI). The locations of cerebral areas corresponding to oral movement control were identified using a smiling motor paradigm. We specified three local networks within the identified areas: the ipsilesional (left) intrahemispheric, contralesional (right) intrahemispheric, and interhemispheric networks. Because functional connectivity is a core feature of resting-state cerebral plasticity, the strength of functional connectivity reflects the intensity of information transfer and the degree of neuronal activity synchronization between cerebral areas (Tokuno et al., 1997; Xiao et al., 2015; Li et al., 2017). We compared the mean intensity of each network connection between patients and healthy subjects, and analyzed the relationship between this intensity and the extent of oral asymmetry. Our study provides insights into the possible cortical etiology of facial asymmetry. Furthermore, by investigating the spatial information of cortical areas, our findings may help surgeons to provide more effective cortical therapeutic interventions to reconstruct facial symmetry.

## Materials and Methods

### Subjects

From 2020 to 2022, 22 patients (13 females, 9 males) with completely unilateral facial nerve injury were enrolled in this study. To eliminate hemisphere-specific effects, 11 patients with right facial nerve injury and 11 patients with left facial nerve injury were investigated in a balanced design. We recruited patients with the specific etiology of postsurgical acoustic neuroma. All patients had no facial muscle contractions on the paralyzed side, and needle electromyography testing verified that there were no signs of action potentials but showed significant fibrillation potential amplitude in the paralyzed facial muscles. The testing results indicate that the muscle fibers still have spontaneous activity and are not completely deformed. Patients scored Grade 1 on Terzise spontaneous activity and ar (Terzis and Noah, 1997). We included patients with (1) denervation time ranging from 12 to 15 months; (2) age from 26 to 55 years; (3) right-handedness as assessed by the Edinburgh Handedness Inventory (Oldfield, 1971); and (4) signed informed consent. Participants were excluded if they had (1) experienced medical treatment for facial dysfunction; (2) acoustic neuroma remains; (3) a confirmed history of mental disease, cerebral disease, or central/peripheral neuropathies; or (4) any contraindications for MRI; (5) droop oral commissure with soft tissue ptosis on the paralyzed side (Terzis and Noah, 1997). This research was approved by the Medical Ethics Committee of the Shanghai Ninth Peopleresearch was approved by thTong University School of Medicine (Shanghai, China; Approval Number SH9H-2021-TK26-1). All procedures were conducted in accordance with the Declaration of Helsinki. For the control group, 15 right-handed age-matched healthy participants (8 females, 7 males) were enrolled.

### Evaluation of Oral Commission Asymmetry

**FIGURE 1:**
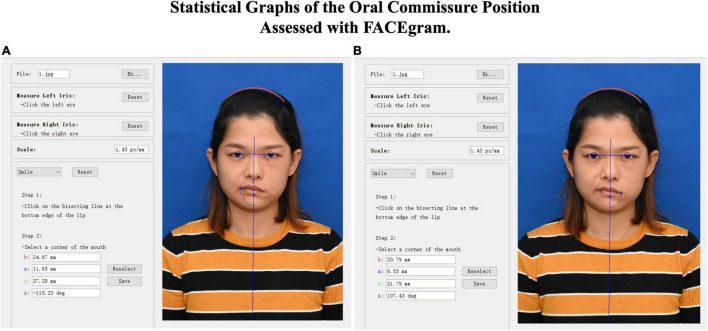
FACEgram was used to quantitatively assess the unaffected **(A)** or paralyzed **(B)** side of the oral commissure at rest. The bilateral pupil and midpoint were used to establish a coordinate system. The o*a-score*”coreinate system. Thfrom the oral commissure perpendicular to the horizontal line at the bottom edge of the lower lip (blue line). The “*b-score*”corefrom the oral commissure perpendicular to the vertical line (red line). The “a*c-score*”corefrom the oral commissure to the bottom edge of the lower lip (green line). The photo shows the static outcome of a 31-year-old woman at 1 year postoperative. The difference in bilateral oral commissure (*c*_*dif*_ = 5.48) was determined by the unaffected (*c_*ua*_* = 27.28) minus the paralyzed (*c_*p*_* = 21.79) oral commissure positions (*c_*dif*_* = *c_*ua*_ - c_*p*_*).

All patients underwent a series of standard recordings, including personal information, photographs, and videos. The Facial Assessment by Computer Evaluation (FACEgram) (Hadlock and Urban, 2012; Chen et al., 2017) software was used to quantitatively assess oral commissure symmetry at rest. The “*c-score*” refers to the measurement from the oral commissure to the bottom edge of the lower lip (*c-line*), while the “*a-score*” and “*b-score*” are the measurements from the oral commissure perpendicular and parallel to the horizontal and vertical lines at the bottom edge of the lower lip (*a-line* and *b-line*). The *a*-, *b*-, and *c*-lines form a right triangle, where *c^2^* = *a^2^* + *b*^2^ (Figure 1). Thus, the “*c-score*” reflects both the vertical and horizontal oral commissure positions. The difference in bilateral oral commissure position (*c*_*dif*_) refers to the oral commissure position of the unaffected side (*c*_*ua*_) minus the paralyzed side (*c*_*p*_; i.e., *c_*dif*_* = *c_*ua*_ - c_*p*_*). A larger value of *c*_*dif*_ indicates greater asymmetry of the oral commissure. All evaluations were performed by one independent observer.

### Motor Task Paradigm

Instructions and a training program were provided before individuals underwent MRI scanning to ensure that all enrolled subjects were able to perform the task. Because of the perioral muscles involved in smiling movements dominate the oral symmetry at resting state. The control individuals were instructed to move their mouth angle up bilaterally to generate a natural smile before relaxing the facial muscles to return to the starting position. The patients with facial nerve lesions received daily paradigm training for 1 week before the scanning session. With the help of a mirror feedback apparatus, patients were asked to both imagine and imitate a natural smile. During MRI, all subjects followed the visual instructions and performed the paradigm. The tasks required voluntary repetitive movements, which were visually guided by emoticons, in each onset epoch of 15 s. Subjects usually performed 15–20 sets of smile tasks in each onset epoch. The baseline was a blank task where participants were asked to focus their eyes on a cross in the middle of the screen. Each session lasted 90 s, which consisted of three repetitions of a 30-s ON-OFF cycle.

### Magnetic Resonance Imaging Protocol

All examinations were performed on the same 3.0 Tesla MR scanner (Prisma, Siemens, Erlangen, Germany) to obtain echo-planar T2*-weighted image volumes (EPI) and transaxial T1-weighted structural images. Functional resting-state data were acquired in one EPI session of 240 volumes. Each subject was instructed to lie down with their eyes closed, avoid any structured thinking, and remain awake. The first three dummy scans were automatically discarded because of magnetic equilibration. A functional image volume was composed of 48 transaxial slices, with a top-down interleaved scanning sequence. The field of view included the whole cerebrum and cerebellum (voxel size = 2 mm × 2 mm × 2 mm, field of view = 188 mm × 188 mm × 96 mm, repetition time = 1.5 s). The task-related fMRI scans used the same parameters and were performed after the resting-state scan. After functional measurements, high-resolution T1-weighted structural images (voxel size = 1 mm × 1 mm × 1 mm) with sagittal reconstruction were acquired.

### Preprocessing of Functional Data (Resting State and Motor Paradigm)

The recruited subjects were affected in both the left and right sides in the present study. Thus, to unify the affected brain side, we flipped some brain images along the anterior–posterior axis to make the left hemisphere in all images be the ipsilesional side. The preprocessing procedures for the functional images from both the resting-state and task-dependent scans were similar. The slicing time procedure was used to adjust the time of image acquisition in one TR according to the hemodynamic equation. Images were then aligned to the reference image and a head motion parameter was generated. Next, covariates such as age, head motion, and cerebrospinal fluid signal were regressed out. Both the task-dependent and resting-state images were then normalized into standard space using the EPI template, and the normalized volumes were smoothed using a 6-mm full-width-at-half-maximum Gaussian kernel. We performed two extra procedures for the resting-state images: detrending and low band-pass (0.01–0.08 Hz) filtering. Other than these two steps, the resting-state and task-dependent images were preprocessed using the same methods.

### Functional Magnetic Resonance Imaging Analysis of Task-Dependent Images

A general linear model was used to generate the statistical maps. Both between- and within-group comparisons were used to reveal cortical activation in the motor task for the two groups. The two-sample *t*-test was also applied to examine differences in motor-task activation between the two groups. The resulting statistical maps were thresholded using false discovery rate (FDR, *p* < 0.05).

### Connectivity Analysis of Resting-State Data

We first localized several regions of interest (ROIs) derived from previous task-dependent fMRI results. According to the voxel size, we selected the largest clusters as the candidates for later analysis. After defining ROIs, a cluster-level time series was extracted by averaging the time series of all voxels with this cluster. Variance (including head motion parameters and white matter signals) was removed from the data using linear regression. Next, weighted brain networks with nodes and edges were established. These ROIs constituted the right hemispheric, left hemispheric, and interhemispheric brain networks. Sparsity was set to 50% to reduce covariance caused by weak connections. Furthermore, Pearson correlation coefficients were calculated between every pair of nodes in the three brain networks. The average connectivity, which represented the mean intensity of connections within the local functional network, was generated for each network. These coefficients were transformed to z-scores using Fisher’s z-transformation. The z-scores of each brain network between the two groups (control vs. facial nerve lesions) were compared separately using a paired *t*-test to detect any significant differences in functional connectivity. In addition, to investigate the relationship between cerebral resting-state functions and oral asymmetry, we performed Pearson correlation analysis on the average functional connectivity of patientsnnectionen every and their clinical data (*c*_*dif*_).

## Results

### Clinical Assessment of Facial Asymmetry

There were no significant differences in demographic details between patients with facial nerve lesions and controls (*p* > 0.05). The details are listed in Table 1.

**TABLE 1:** Patients demographics.

Variable	Patient group	Health controls	*P*
No.	22	15	
Sex			
Female	13	8	
Male	9	7	0.73^a^
Paralyzed side			
Left	11	N/A	
Right	11	N/A	
Age (year)			
Mean ± SD	38.86 ± 6.92	35.80 ± 9.27	0.26^b^
Range	26–55	19–53	
Evolution (month)			
Mean ± SD	13.41 ± 1.14	N/A	
Range	12–15	N/A	

*^a^Chi-square test; ^b^Two sample t-test.*

In the patients with facial nerve lesions, the *c*_*p*_ (26.26 ± 2.15 mm, range 21.59–29.33 mm) was significantly smaller than the *c*_*ua*_ (35.67 ± 4.24 mm, range 27.27–44.19 mm) at rest. The mean *c*_*dif*_ was 9.41 ± 3.48 mm (range 4.72–15.48 mm).

### Motor Task Data

**FIGURE 2:**
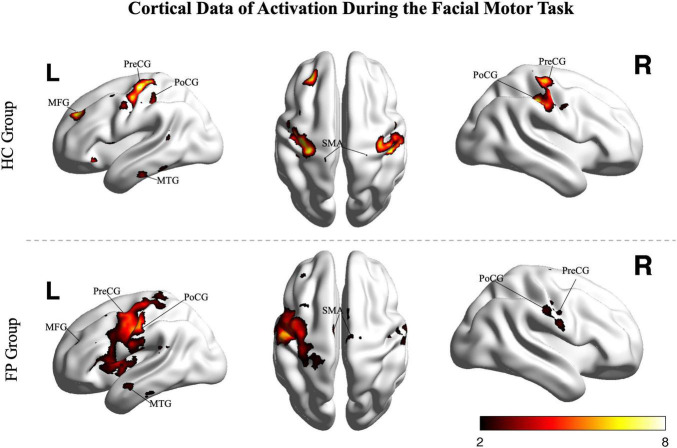
Random effects group analysis of the facial smiling task. Activations (one sample *t*-test, *p* < 0.05, FDR corrected) in response to blocked (15 s) movement of the bilateral mouth angle are shown superimposed on a template cortex. Images from the control group (*n* = 15) are shown in the upper part of the figure, while images from the patients (*n* = 22) are shown in the lower part. PoCG, postcentral gyrus; PreCG, precentral gyrus; SMA, supplementary motor area; MFG, middle frontal gyrus; MTG, middle temporal gyrus; R, right; L, left; FP, facial paralysis patients; HC, healthy controls. Color bar shows the *t*-value.

The smile task evoked highly significant activations in both the patients and controls (*p* < 0.05, FDR corrected, Figure 2); however, activation patterns were significantly different between the two groups.

In the control group, the smile task mainly recruited cortical areas in the precentral gyrus (bilateral primary motor area), postcentral gyrus (somatosensory motor area), middle frontal gyrus, superior frontal gyrus (supplementary motor area), and middle temporal gyrus. The main recruited subcortical areas were the insula, putamen, basal ganglia, thalamus, and lobules VI and VIII of the cerebellum.

**FIGURE 3:**
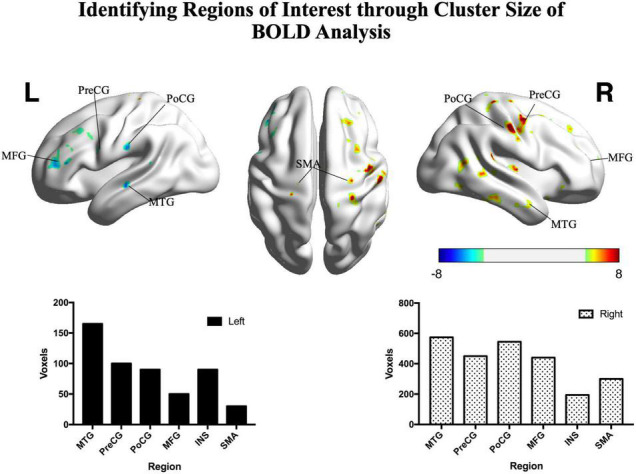
Significant differences between patients and controls in the motor task. Hot colors represent areas that were significantly greater in the controls (*n* = 15) compared with patients (*n* = 22), while cold colors represent areas that were significantly greater in the patients compared with controls (two sample *t*-test, *p* < 0.05, FDR corrected). The histograms show significant clusters in the right and left hemispheres, respectively. PoCG, postcentral gyrus; PreCG, precentral gyrus; SMA, supplementary motor area; MFG, middle frontal gyrus; MTG, middle temporal gyrus; INS, insula; R, right; L, left. Color bar shows the *t*-value.

In patients, the ipsilesional hemisphere (left side) showed a similar activation pattern to that of the controls, but the activation was greater. In contrast, the contralesional hemisphere (right side) showed relatively weak activation in the precentral, postcentral, and superior frontal gyri. The activation images of the patients and the differences between groups are shown in Figure 3.

### Functional Connectivity

**FIGURE 4:**
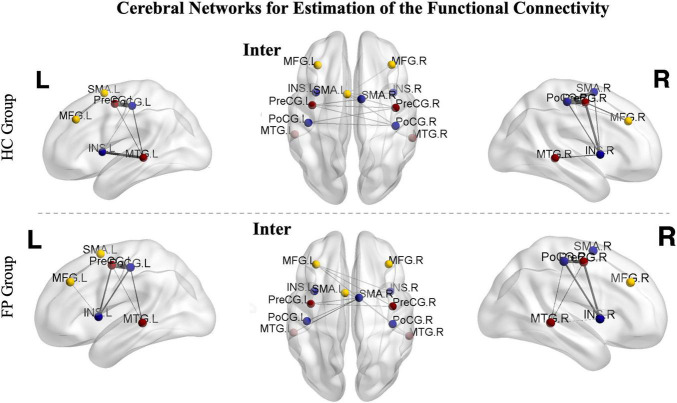
Cerebral networks for the estimation of functional connectivity. The identified areas constitute the right hemispheric, left hemispheric, and interhemispheric networks. The nodes represent the peak activated locations, and the edges represent the strengths of connectivity. The images from controls are shown in the upper part of the figure, while images from the patients are shown in the lower part. Sparsity was set to 50%. PoCG, postcentral gyrus; PreCG, precentral gyrus; SMA, supplementary motor area; MFG, middle frontal gyrus; MTG, middle temporal gyrus; INS, insula; R, right; L, left; FP, facial paralysis patients; HC, healthy controls.

To reduce the number of tests and focus on our primary research aim, we selected the top six functional cerebral areas that were most activated during the smiling motor task. We then estimated the functional connectivity within the left intrahemispheric, right intrahemispheric, and interhemispheric networks and compared them using paired *t*-tests (Figure 4).

In the patient group, functional connectivity of the ipsilesional network (left, *r* = 0.65 ± 0.10) was significantly higher than that of the contralesional network (right, *r* = 0.48 ± 0.11, *p* < 0.05). In the control group, there was no significant difference between the left and right networks (left vs. right, 0.59 ± 0.08 vs. 0.58 ± 0.07, *p* > 0.05).

**FIGURE 5:**
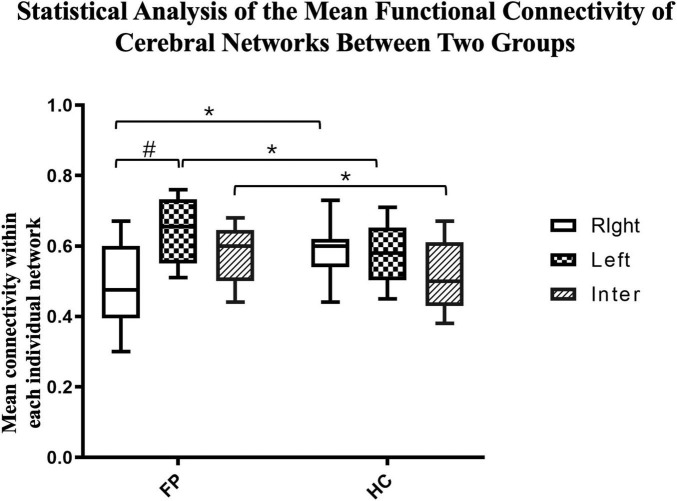
Statistical analysis of the mean functional connectivity of cerebral networks between the two groups. The mean functional connectivity in the left, right, and inter-hemispheric networks of patients (*n* = 22) and controls (*n* = 15) are shown. These network-specific averaged z-score values (*via* Fisher’s z-transformation) were then tested for differences both within and between groups. *p < 0.05 between groups; ^#^*p* < 0.05 within groups; paired *t*-test.

We then further investigated the differences between the two groups. The connectivity of both the left (*r* = 0.65 ± 0.10) and interhemispheric (*r* = 0.58 ± 0.08) networks were significantly higher in the patient group than in the control group (right = 0.58 ± 0.07, interhemispheric = 0.52 ± 0.10, *p* < 0.05). However, the connectivity in the right network was significantly lower in the patient group than in the control group (patient vs. control, 0.48 ± 0.11 vs. 0.58 ± 0.07, *p* < 0.05) (Figure 5).

### Clinical Correlations

**FIGURE 6:**
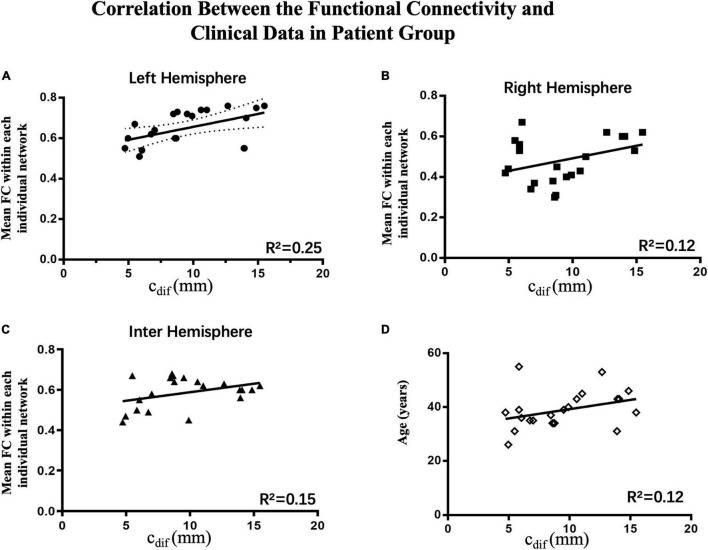
Correlations between connectivity and clinical data in the patient group (*n* = 22). The Pearson correlations of the average connectivity and age of individual patients with oral asymmetry are shown. **(A)** There was a significant correlation between functional connectivity in the ipsilesional network (left hemisphere, *R*^2^ = 0.25, *p* < 0.05) and *c*_*dif*_; increased connectivity correlated with greater oral asymmetry. **(B–D)** Contralesional hemispheric connectivity (right hemisphere, *R*^2^ = 0.12, *p* > 0.05), interhemispheric connectivity (*R*^2^ = 0.13, *p* > 0.05), and age (*R*^2^ = 0.12, *p* > 0.05) were not significantly correlated with *c*_*dif*_.

To investigation correlations between oral commissure asymmetry (*c*_*dif*_) and connectivity in the right, left, and interhemispheric networks, Pearson regression analysis was performed on each individual patient. To eliminate the influence of age on the results, patient age was also examined in this model. There was a significant correlation between the functional connectivity of the ipsilesional network (left, *R*^2^ = 0.25, *p* < 0.05) and *c*_*dif*_. In contrast, age (*R*^2^ = 0.12, *p* > 0.05), the contralesional network (right, *R*^2^ = 0.12, *p* > 0.05), and interhemispheric connectivity (*R*^2^ = 0.15, *p* > 0.05) were not significantly correlated with *c*_*dif*_ (Figure 6). These results indicate that greater asymmetry of the oral commissure is related to stronger connectivity in the ipsilesional hemisphere. Thus, the increased connectivity in the ipsilesional hemisphere may result in hypertonic facial muscles on the unaffected side at rest.

## Discussion

Facial paralysis caused by a facial nerve lesion can result in a series of difficult consequences for patients. For example, asymmetrical facial expressions can lead to depression and social barriers (Baugh et al., 2013). Static asymmetry is one of the major complaints of some patients, while other patients have only slight oral commissure asymmetry. Even with the same etiology of facial nerve injury and similar onset times and ages, patients experienced very different levels of oral commissure asymmetry at rest in the current study. Consistent with recent studies, in patients who underwent total facial nerve transection approximately 1 year previously, we revealed hyperactivation of ipsilesional cerebral areas, which innervate the unaffected facial muscles during smiling. In contrast, the contralesional hemisphere, which innervates the paralyzed facial muscles, had weakened activation. Notably, we also demonstrated that increased functional connectivity in the ipsilesional hemisphere was significantly correlated with oral commissure asymmetry. A summary of our results is as follows: (a) the network within the ipsilesional hemisphere, which consists of cerebral areas corresponding to the facial motor task, presented a maladaptive increase in connectivity; (b) connectivity within the contralesional network was consequently reduced because of interhemispheric inhibition from the opposite hemisphere together with long-term disuse of the paralyzed facial muscles; and (c) interhemispheric connectivity, which was likely inhibitive because of the interhemispheric inhibition effect, was significantly increased in patients.

Using positron emission tomography, transcranial magnetic stimulation, and fMRI, morphometric analysis has revealed that there is widespread cerebral reorganization, including motor and somatosensory reorganization, after facial nerve injury (Rijntjes et al., 1997; Bitter et al., 2011; Klingner et al., 2014; Rottler et al., 2014). This reorganization mainly occurs in the bilateral primary motor cortex, sensorimotor cortex, middle temporal gyrus, promotor cortex, cingulate motor area, supplementary motor area, supramarginal gyrus, precuneus, and subcortical areas including the thalamus, insula, and cerebellum. Consistent with these previous studies, we found that the areas with the most changes were localized in regions such as the middle temporal gyrus, bilateral primary motor cortex, sensorimotor cortex, middle frontal gyrus, supplementary motor area, and insula. After facial nerve lesions, changes in these areas are primarily responsible for mediating facial movement, processing sensorimotor information, higher order processing, and cognitive integration in learning and memory (Downar et al., 2002; Hickmott and Steen, 2005; Klingner et al., 2011). This reorganization plays a crucial role in determining how well a patient adapts after a facial nerve lesion.

Facial palsy involves a complete motor deefferentation (i.e., a loss of motor output). In contrast, deefferentation from the innervated brain area to the peripheral facial muscles as the result of a facial nerve lesion cause a motor–sensory mismatch error between the desired movement (motor) and the perceived executed movement (somatosensory feedback) (Klingner et al., 2014, 2019). This mismatch is a form of adaptation *via* cerebral plasticity. Thus, when patients perform facial motor tasks, more attention and visualization of the movements might occur compared with healthy individuals (Klingner et al., 2019; Ling et al., 2021). This results in the following errors: the commands of the ipsilesional functional area fail to execute and there is hypertonicity of the unaffected facial muscles. Our previous study suggested that hypertonicity of facial muscles on the unaffected side is an important factor that affects facial symmetry (Ma et al., 2022). In the present study, the activation of ipsilesional sensorimotor areas, which innervate the unaffected facial muscles, was higher than that of healthy subjects. A plausible explanation might be that there is increased neural activation in multiple ipsilesional cerebral areas of patients (Ling et al., 2021). Previous group analysis studies have reported that activation in the primary motor cortex, putamen, thalamus, supplementary motor area, sensorimotor cortex, cerebellum, and insula is significantly increased during movement execution (Rijntjes et al., 1997; Xiao et al., 2015; Calistri et al., 2021). Moreover, increased activation in the frontal lobe, supplementary motor area, and insula plays an important role in the increased awareness of imminent paretic facial expressions in the ipsilesional pathway (Kobayashi et al., 2003; Salardini et al., 2012; Finis et al., 2013). This persistent mismatch is a powerful driver of sensorimotor adaptation, and mismatch error is then positively reinforced (Klingner et al., 2021), leading to more hypertonicity of the unaffected facial muscles. Based on the interhemispheric competition model, hyperactivation of the ipsilesional hemisphere has an inhibitory effect on activation of the contralesional hemisphere *via* callosal pathways (Daskalakis et al., 2002; Ginatempo et al., 2019; Carson, 2020). In this model, the hyperactivation is positively related to the extent of inhibition, and thus to the decreasing activation of the contralesional hemisphere. The interhemispheric inhibition effect and its relationship with motor deficits have been demonstrated in previous studies of patients with paretic upper limbs (Lefebvre et al., 2014; Palmer et al., 2019). Because of the similarities and differences in the anatomical structures of the facial and spinal nerves. The changes of cerebral plasticity caused by facial nerve lesions are more extensive compared to spinal nerve lesions (Hwang et al., 2015). These processes have been suggested to be maladaptive motor-sensory plasticity, which is correlated with the severity of facial palsy (Rijntjes et al., 1997).

Abnormal functional connectivity is a core feature of maladaptive cerebral plasticity in patients with facial paralysis (Li et al., 2017), and has been reported to correlate with facial nerve function (Albert et al., 2009; Hampstead et al., 2011). In patients with Bell’s palsy, decreased functional connectivity in the contralesional motor and somatosensory networks is related to sensorimotor integration and modulation in the early stages (Carter et al., 2010; van Meer et al., 2010; Klingner et al., 2014, 2019). Later, the strength of functional connectivity increases, reflecting the recovery of facial function. These changes are associated with the clinical severity of Bell’s palsy, and are derived from asymmetrical compensation (Han et al., 2019; Wu et al., 2019). The strength of functional connectivity increases to a stable level until recovery stabilizes (Klingner et al., 2012). Moreover, increased ipsilesional connectivity, such as of the ipsilesional anterior cingulate cortex with the primary motor cortex, supplementary motor area, premotor, bilateral dorsolateral prefrontal cortex, midcingulate cortex, and sensorimotor cortex, strengthens the movements of facial expression muscles in patients with Bell’s palsy (Hu et al., 2015). Furthermore, increased amplitude of low-frequency fluctuation values have been reported in the ipsilesional insula, which might reflect a hypercompensation of spontaneous neuronal activity in the resting state (Wu et al., 2019).

In the current study, the increased connectivity of facial expression-related areas in the ipsilesional hemisphere indicate an attempt to resolve sensory-motor discrepancies by modulating cerebral functional connectivity, and were correlated with oral commissure asymmetry. The ipsilesional hemisphere has a positive inhibitory influence on pyramidal tract cells *via* callosal projections to the contralesional hemisphere. When more positive inhibitory information flows through the corpus callosum, the connectivity of the interhemispheric network is increased (Ferbert et al., 1992; Hübers et al., 2008). We found increased connectivity of the interhemispheric network in patients with facial nerve lesions. The relatively long-term denervation and transneuronal degeneration of the paralyzed facial muscles, together with interhemispheric inhibition from the opposite hemisphere, induced significantly decreased activation in contralesional hemisphere areas. Maladaptive processes occurred, and the area of facial representation was likely overtaken by neighboring cortical areas (e.g., hand or tongue representation areas) (Sanes et al., 1990; Rödel et al., 2004; Lotze et al., 2006). Functional connectivity within the contralesional hemisphere was significantly reduced. This decrease reflects an unsuccessful sensory-motor adaptation process, caused by the inability—and interhemispheric inhibitory influences—of contralesional cerebral areas to resolve the sensory-motor mismatch. However, these changes did not significantly correlate with oral commissure asymmetry. These results indicate that maladaptive changes in cerebral plasticity occur in patients with long-term unilateral facial nerve lesions. The findings also extend our understanding of sensory-motor interactions in response to mismatched signals in the later stages of injury. Our study provides insights into the mechanisms of facial asymmetry in central nervous system plasticity. And the results can be translated into a connectivity-based targeting strategy for focal cerebral stimulation. Early interventions, such as transcranial magnetic stimulation, transcranial direct current stimulation, or acupuncture, might be beneficial therapeutic options for improving facial symmetry.

## Limitations

A prospective study on the dynamic and time-dependent correlations between functional connectivity and oral commissure asymmetry would be a more robust way to investigate the mechanisms of facial asymmetry in central nervous system plasticity. Current knowledge supports the view that the mismatch leads to hypertonicity of the unaffected facial muscles. The maladaptive plasticity on the other hand consolidates the dysfunction of facial nerve in the later stage. We believe this is a highly interrelated and mutually reinforcing process. However, we could not specify the causal relationship between the facial asymmetry and maladaptive plasticity. Furthermore, patients with facial palsy experience oral commissure asymmetry both at rest and while smiling. However, we have found that large variations in oral commissure asymmetry occur during smiling, even in a single patient at different times or in different emotional states. A unified standard for measuring oral asymmetry while smiling is thus needed. We have also tried to test the strength and action potentials of facial muscles, but there was again large variation. In future studies, we will investigate correlations between the hypertonicity of facial muscles and cerebral plasticity in patients with facial palsy.

## Conclusion

A long-term course of facial nerve transection induced widespread sensory-motor maladaptations and a mismatch in cerebral reorganization. There were extensive changes in activation patterns and functional connectivity of patients with facial nerve lesions. Increased connectivity of smiling-related areas in the ipsilesional hemisphere correlated with oral commissure asymmetry. We suggest that this modulated functional connectivity indicates an attempt to compensate for facial asymmetry proprioception at rest. Our study provides valuable spatial information for future clinical cerebral interventions and rehabilitative processes that promote facial symmetry.

## Data Availability Statement

The original contributions presented in the study are included in the article/supplementary material, further inquiries can be directed to the corresponding author/s.

## Ethics Statement

The studies involving human participants were reviewed and approved by the Medical Ethics Committee of the Shanghai Ninth People’s Hospital, Shanghai Jiao Tong University School of Medicine (Shanghai, China; Approval Number SH9H-2021-TK26-1). The patients/participants provided their written informed consent to participate in this study. Written informed consent was obtained from the individual(s) for the publication of any potentially identifiable images or data included in this article.

## Author Contributions

HM performed wrote the manuscript and drew the figures. Y-LZ, W-JW, and GC provided advises of modification. QL provided the fMRI scanning protocol. Y-CL performed the data analysis and submitted the manuscript. WW provided the main design of this study. All authors contributed to the article and approved the submitted version.

## Conflict of Interest

QL was employed by the Siemens Healthineers Ltd. The remaining authors declare that the research was conducted in the absence of any commercial or financial relationships that could be construed as a potential conflict of interest.

## Publisher’s Note

All claims expressed in this article are solely those of the authors and do not necessarily represent those of their affiliated organizations, or those of the publisher, the editors and the reviewers. Any product that may be evaluated in this article, or claim that may be made by its manufacturer, is not guaranteed or endorsed by the publisher.
